# Supplementary material for: Fine-tuning citrate synthase flux potentiates and refines metabolic innovation in the Lenski evolution experiment
Source: eLife. 2015 Oct 14;4:e09696. doi: 10.7554/eLife.09696 (PMC4718724; doi:10.7554/eLife.09696)
Supplement: Figure 1—source data 1. — DOI: http://dx.doi.org/10.7554/eLife.09696.004 [file elife-09696-fig1-data1.docx]

**Figure 1–source data 1. Details for all *gltA* alleles in this study**

| **Allele** | **Position** | **Mutation** | **Effect** | **Source** |
| --- | --- | --- | --- | --- |
| *gltA1* | 734,488 | C→T | A258T (GCT→ACT) | LTEE 25K, 33K–40K (ZDB478, ZDB483, all Cit^+^) |
| *gltA2-1* | 735,273 | A→G | 14 bases upstream | LTEE 33K–33.5K |
| *gltA2-2* | 735,109 | C→T | A51T (GCA→ACA) | LTEE 36K |
| *gltA2-3* | 734,938 | C→T | V108M (GTG→ATG) | LTEE 36K |
| *gltA2-4* | 734,890 | C→T | A124T (GCT→ACT) | LTEE 38K–40K (ZDB107, REL10979) |
| *gltA2-5* | 734,833 | C→T | A143T (GCG→ACG) | LTEE 34K |
| *gltA2-6* | 734,805 | A→G | V152A (GTT→GCT) | LTEE (ZDB96) |
| *gltA2-7* | 734,775 | G→A | A162V (GCG→GTG) | LTEE 34K (ZDB83) |
| *gltA2-8* | 734,533 | C→T | G243S (GGC→AGC) | LTEE 36K |
| *gltA2-9* | 734,514 | G→A | P249L (CCG→CTG) | LTEE 33.5K |
| *gltA2-R* | 735,441 | T→G | 182 bases upstream | REGRES |

**Allele**: Designation used in this study for this mutation.

**Position**: Mutated position in ancestral REL606 genome coordinates (GenBank: NC_012967.1).

**Mutation**: Base change caused by this mutation on the top strand of the genome.

**Effect**: For mutations within the protein coding sequence, the abbreviated amino acid change is shown and the codon change is given in parentheses with the mutated base underlined. For noncoding mutations, the number of bases upstream of the protein start codon is shown.

**Source**: Experiment in which the allele was found (LTEE or REGRES). LTEE population samples are indicated by generation (e.g., 36K is the 36,000-generation sample), and specific clonal isolates containing the evolved allele are shown in parentheses (e.g., ZDB83).
