# Supplementary material for: Fine-tuning citrate synthase flux potentiates and refines metabolic innovation in the Lenski evolution experiment
Source: eLife. 2015 Oct 14;4:e09696. doi: 10.7554/eLife.09696 (PMC4718724; doi:10.7554/eLife.09696)
Supplement: Figure 1—source data 2. — DOI: http://dx.doi.org/10.7554/eLife.09696.005 [file elife-09696-fig1-data2.docx]

**Figure 1—source data 2. Mutations used to track clade dynamics in the LTEE**

| **Generation** | **Sample** | ***metL* (C1)** | ***gltA1* (C3)** | ***iclR* (C2+C3)** |
| --- | --- | --- | --- | --- |
| 20,000 | REL8595M | 47.7% [39.5%–56.0%] | 0.0% [0.0%–2.4%] | 46.6% [37.8%–55.5%] |
| 25,000 | REL10177 | 19.9% [13.6%–27.4%] | 41.3% [32.4%–50.6%] | 69.8% [62.0%–76.8%] |
| 30,000 | REL10381 | 14.7% [9.2%–21.8%] | 0.0% [0.0%–2.9%] | 78.8% [68.2%–87.1%] |
| 31,500 | REL10455 | 17.2% [11.7%–23.9%] | 0.0% [0.0%–2.4%] | 87.0% [81.0%–91.7%] |
| 32,000 | REL10467 | 50.2% [43.2%–57.3%] | 1.6% [0.3%–4.5%] | 43.0% [35.8%–50.5%] |
| 32,500 | REL10479 | 61.9% [55.5%–68.0%] | 0.0% [0.0%–1.9%] | 36.7% [28.9%–45.1%] |
| 33,000 | REL10491 | 42.7% [36.4%–49.2%] | 14.3% [9.7%–20.0%] | 53.5% [45.7%–61.2%] |
| 33,500 | REL10591 | 0.0% [0.0%–1.2%] | 98.5% [94.6%–99.8%] | 100.0% [97.6%–100.0%] |
| 34,000 | REL10593 | 0.0% [0.0%–2.1%] | 97.7% [93.5%–99.5%] | 100.0% [98.0%–100.0%] |
| 36,000 | REL10810 | 0.0% [0.0%–2.0%] | 96.2% [91.3%–98.7%] | 100.0% [98.0%–100.0%] |
| 38,000 | REL10884 | 0.0% [0.0%–3.6%] | 97.1% [91.7%–99.4%] | 99.2% [95.4%–100.0%] |
| 40,000 | REL10969 | 0.0% [0.0%–2.4%] | 99.0% [94.7%–100.0%] | 100.0% [96.9%–100.0%] |

Three major clades (C1, C2, and C3) diverged before 20,000 generations in the focal Ara–3 population of the Lenski LTEE. For each whole-population sample archived at the specified time point, we used metagenomic DNA sequencing to estimate the frequencies of the mutations that are characteristic of each clade (e.g., C2) or superclade (e.g., C2+C3). Illumina reads were aligned by the *breseq* pipeline to the reference genome and the counts of reads with the variant base versus the reference base were used to estimate a binomial 95% confidence interval for the frequency of that mutation in each sample. This information was used to construct **Figure 1**B. In particular, it shows that an alternative clade (C2) proportionally increased in frequency at 30,000 generations when the clade with *gltA1* that eventually became Cit^+^ (C3) decreased. Note that the reported frequencies of each mutation were estimated independently, based on a distinct subset of Illumina reads that aligned to that particular location in the reference genome. Details for each mutation that was profiled are as follows: *metL* (position 4,111,342; mutation C→T), *gltA1* (position 734,488; mutation C→T), *iclR* (position 4,201,958; mutation A→C). Positions are relative to the ancestral REL606 strain genome (GenBank: NC_012967.1).
