# Supplementary material for: Fine-tuning citrate synthase flux potentiates and refines metabolic innovation in the Lenski evolution experiment
Source: eLife. 2015 Oct 14;4:e09696. doi: 10.7554/eLife.09696 (PMC4718724; doi:10.7554/eLife.09696)
Supplement: Supplementary file 1. — DOI: http://dx.doi.org/10.7554/eLife.09696.015 [file elife-09696-supp1.docx]

**Supplementary File 1. *E. coli* strains and plasmids used in this study**

| **Strain** | **Relevant characteristic** | **Reference** |
| --- | --- | --- |
| REL607 | LTEE ancestor, Ara^+^, Cit^–^ | (Lenski et al., 1991) |
| ZDB564 | 31,500 generation Ara­–3 clone, Ara^–^, Cit^+^ | (Blount et al., 2012) |
| ZDB765 | ZDB564 Ara^+^, Cit^+^ | This work |
| ZDB706 | ZDB564 Ara^–^, Cit^–^ revertant | This work |
| EQ1129 | ZDB706 Ara^+^, Cit^–^ | This work |
| EQ1207 | ZDB564 Ara^+^, *gltA^wt^*, Cit^+^ | This work |
| EQ1208 | ZDB706 Ara^+^ *gltA^wt^*, Cit^–^ | This work |
| ZDB478 | 25,000 generation Ara–3 clone, Ara^–^, Cit^–^ | (Blount et al., 2012) |
| EQ1159 | ZDB478 Ara^+^, Cit^–^ | This work |
| EQ1158 | ZDB478 Ara^–^ *gltA^wt^*, Cit^–^ | This work |
| ZDB483 | 25,000 generation Ara–3 clone, Ara^–^, Cit^–^ | (Blount et al., 2012) |
| EQ1163 | ZDB483 Ara^+^, Cit^–^ | This work |
| EQ1151 | ZDB483 Ara^–^, *gltA^wt^*, Cit^–^ | This work |
| EQ119 | REL607 Ara^+^, *dctA**, Cit^–^ | (Quandt et al., 2014) |
| EQ120 | REL607 Ara^+^, *dctA*,* *gltA1*, Cit^–^ | This work |
| EQ121 | REL607 Ara^+^, *dctA**, *gltA1*, *gltA2-R*, Cit^–^ | This work |
| EQ128 | REL607 Ara^+^, *iclR*, Cit^–^ | This work |
| EQ129 | REL607 Ara^+^, *arcB*, Cit^–^ | This work |
| EQ131 | REL607 Ara^+^, *iclR*, *arcB,* Cit^–^ | This work |
| **Plasmid** | **Relevant characteristic** | **Reference** |
| gltA1-pKO3 | pKO3 derivative for introduction of *gltA1*(A258T) mutation | This work |
| gltA^wt^-pKO3 | pKO3 derivative for introduction of *gltA^wt^* (A258) mutation | This work |
| iclR-pKO3 | pKO3 derivative for introduction of *iclR* (L201R) mutation | This work |
| arcB-pKO3 | pKO3 derivative for introduction of *arcB* (Q79L) mutation | This work |
| pCitT | pSB3K3 derivative containing *rnk*-*citT* mutation | (Quandt et al., 2014) |
| His_6_-gltA-pET28b | pET28b derivative for inducible expression of His_6_-WT GltA protein | This work |
| His_6_-gltA(A258T)-pET28b | pET28b derivative for inducible expression of His_6_-GltA(A258T) protein | This work |
| His_6_-gltA(A258T, A162V)-pET28b | pET28b derivative for inducible expression of His_6_-GltA(A258T, A162V) protein | This work |
| His_6_-gltA(A258T, A124T)-pET28b | pET28b derivative for inducible expression of His_6_-GltA(A258T,A124T) protein | This work |
